# Dental Surgery

**Published:** 1887-04

**Authors:** 


### DENTAL SURGERY.

Dr. Norman W. Kingsley, president of the Dental Society of the State of New York, says:

"There are three kinds of operations involved in the planting of teeth, and they are known as replanting, transplanting and implanting. Replanting is the operation where the teeth are removed and replaced in the same mouth and in the same sockets ; transplanting consists in taking the teeth from another mouth and placing them in sockets from which the teeth or roots have recently been extracted; and implanting is the process of making new sockets in the solid bone or where the teeth have long been extracted and the bone has become solid, and where the teeth from other mouths or the same mouths are implanted in the solid bone. Transplanting and replanting are old methods, one of the earliest successes in them being that of John Hunter, some hundred years ago. In his experiments he found that a tooth recently removed appeared to grow when transferred to the comb of a cock, and this and similar experiments led him to conclude that a tooth would grow in firmly when surrounded by living tissues and his success in many instances led to the process of transplanting teeth. Upon this was based the plan of removing bad teeth and putting in their place fresh teeth obtained from the mouth of another person. This tooth was placed in the newly made socket and in more recent times the tooth has been removed, cleaned, the pulps removed, one or both, and then the tooth replaced in the same socket. This latter is particularly the case where the teeth have been knocked out by accident or injury and they have adhered.

"Implanting is of recent date, and probably originated with Dr. Younger, of California. He conceived the idea of making an entirely new socket in the solid bone and implanting there a tooth from the same mouth or from that of another person. As this process is in its experimental stage, it is not possible to tell yet, how long teeth so implanted will remain firm in the sockets, but apparently satisfactory results have been obtained; the teeth in instances thus far tried, when manipulated by skilled dentists, have taken vigorously to their new environments, and seemingly satisfactory results have ensued.

"This process is based on the theory that if the membrane which originally surrounded the tooth still adheres to it, although it has become dried even, it will be revitalized when the tooth is implanted in its new socket."

